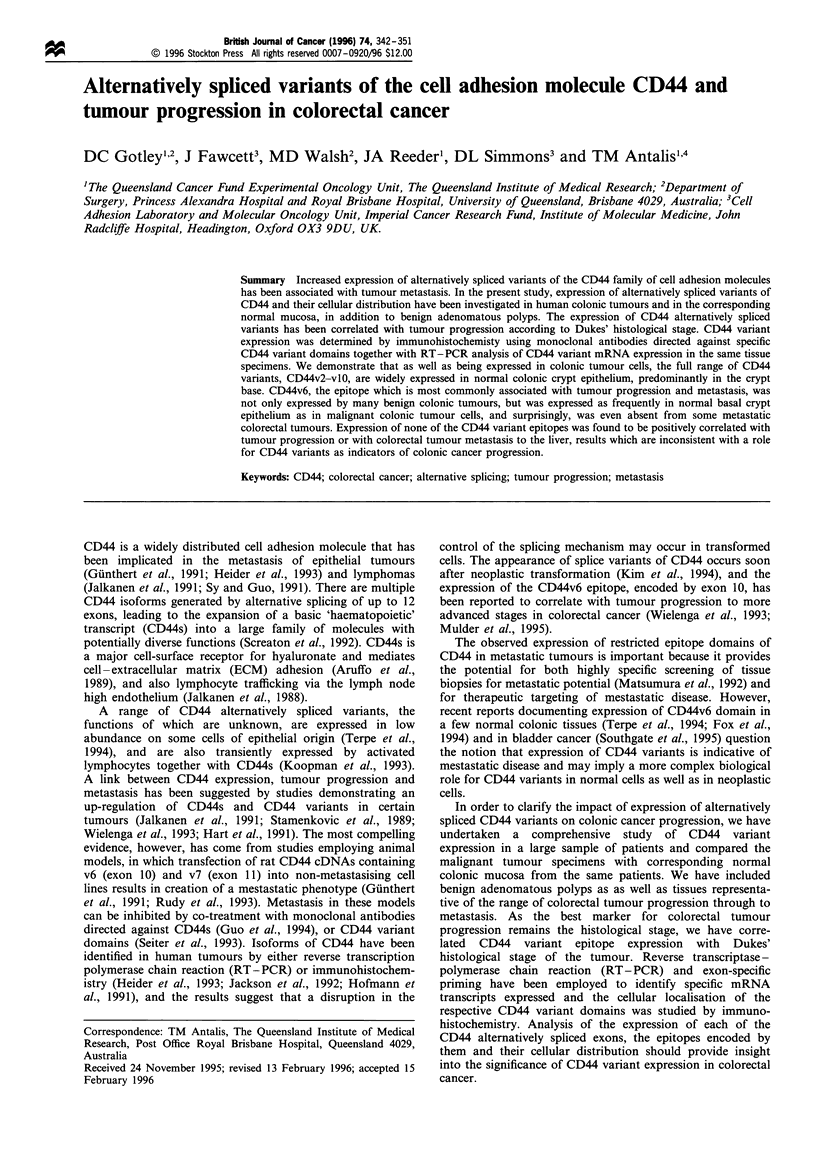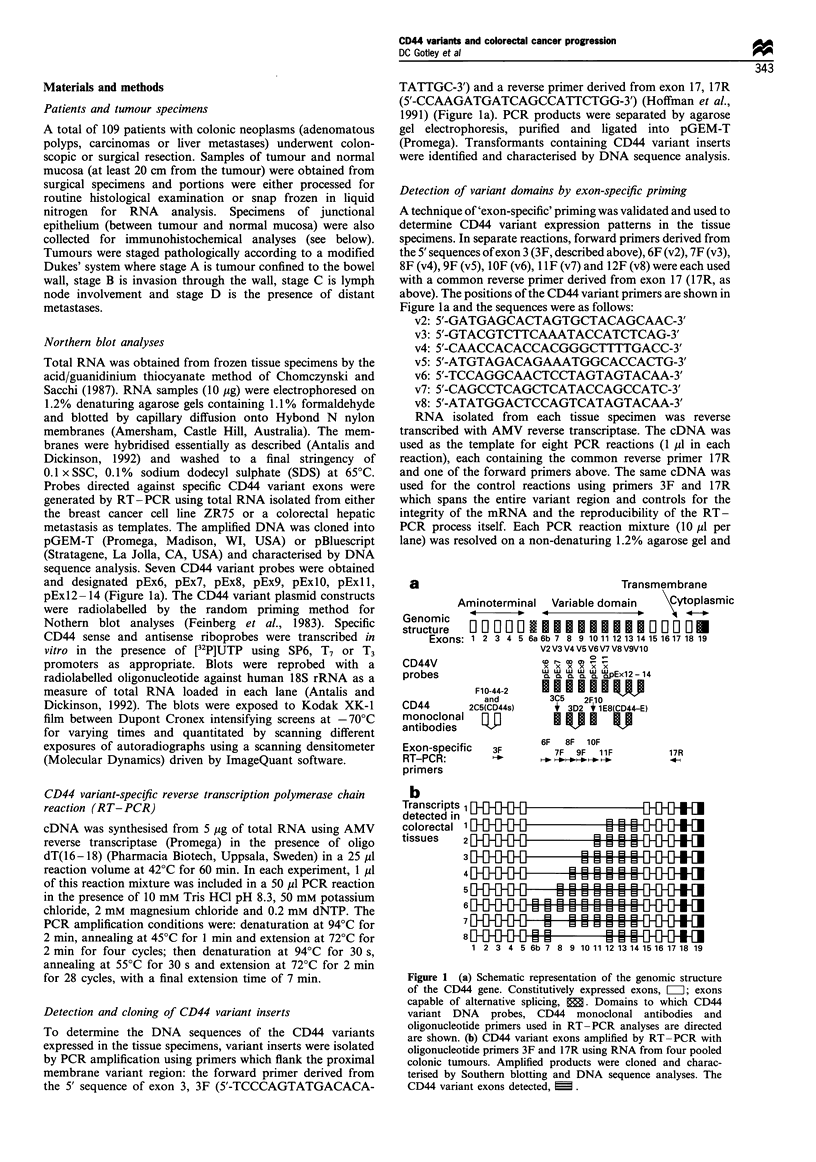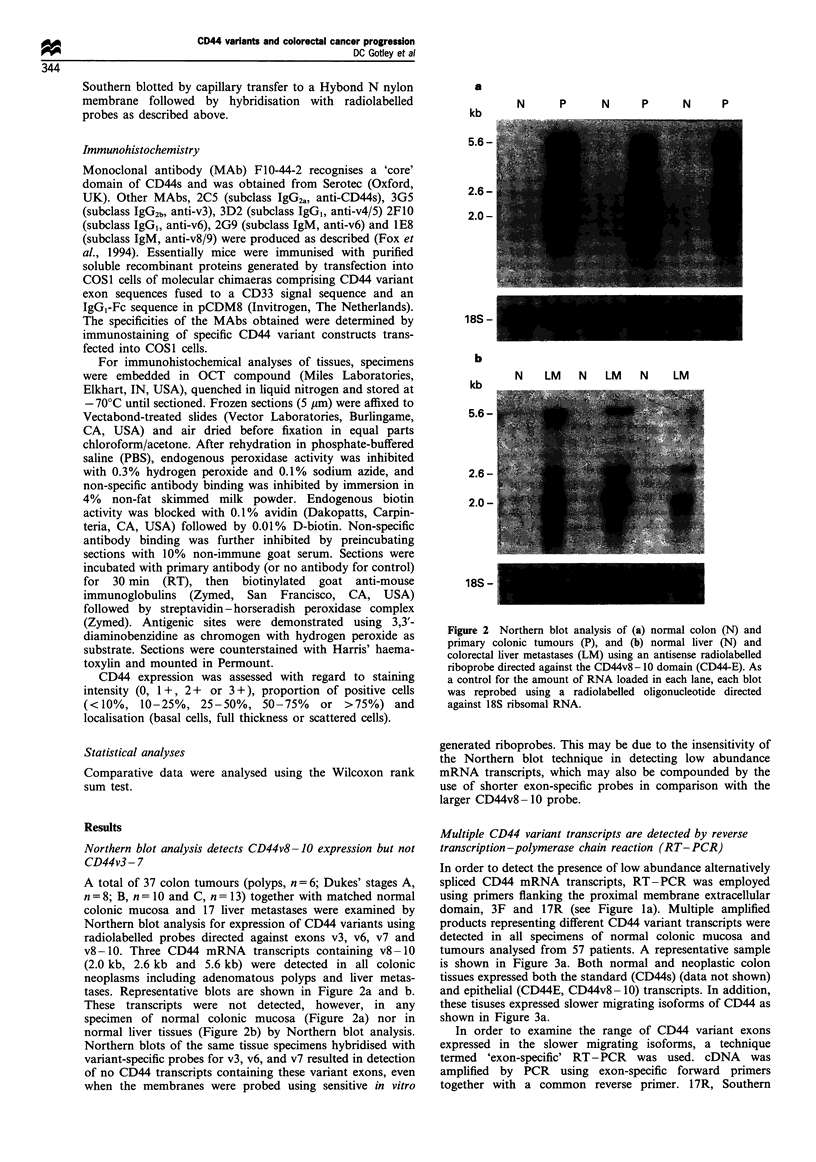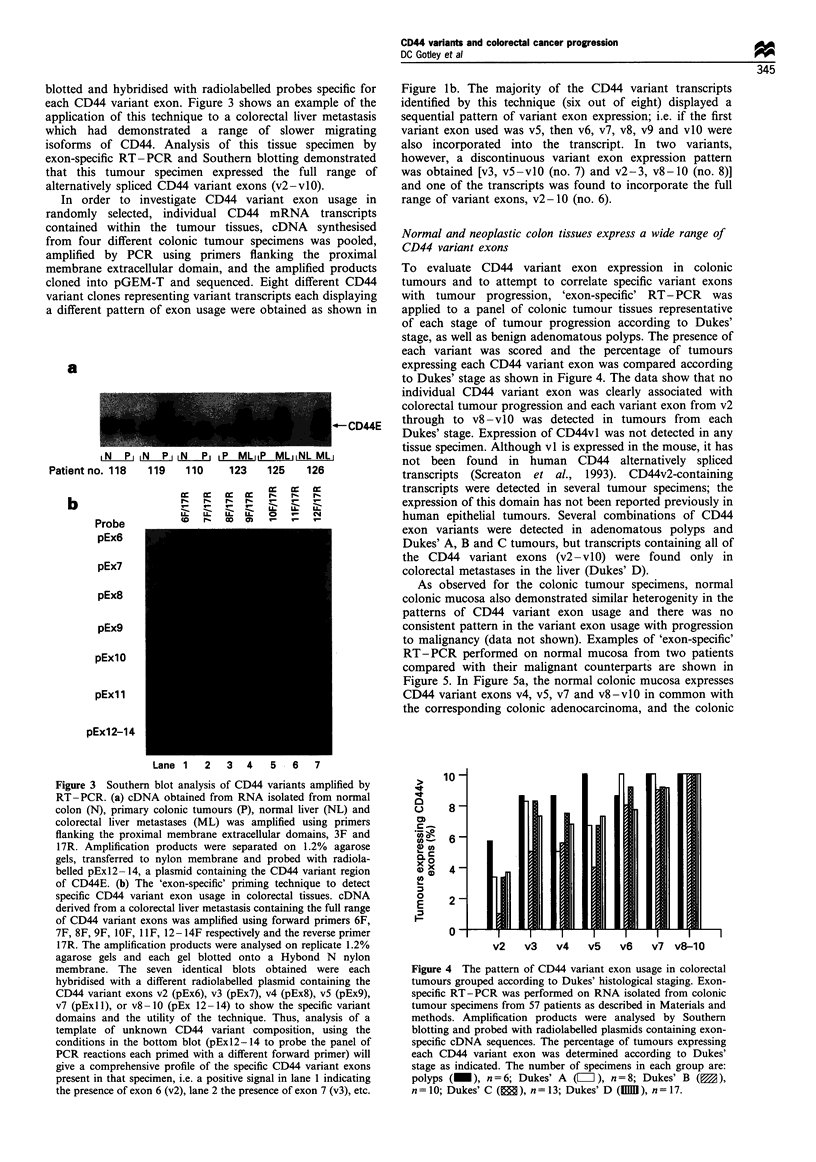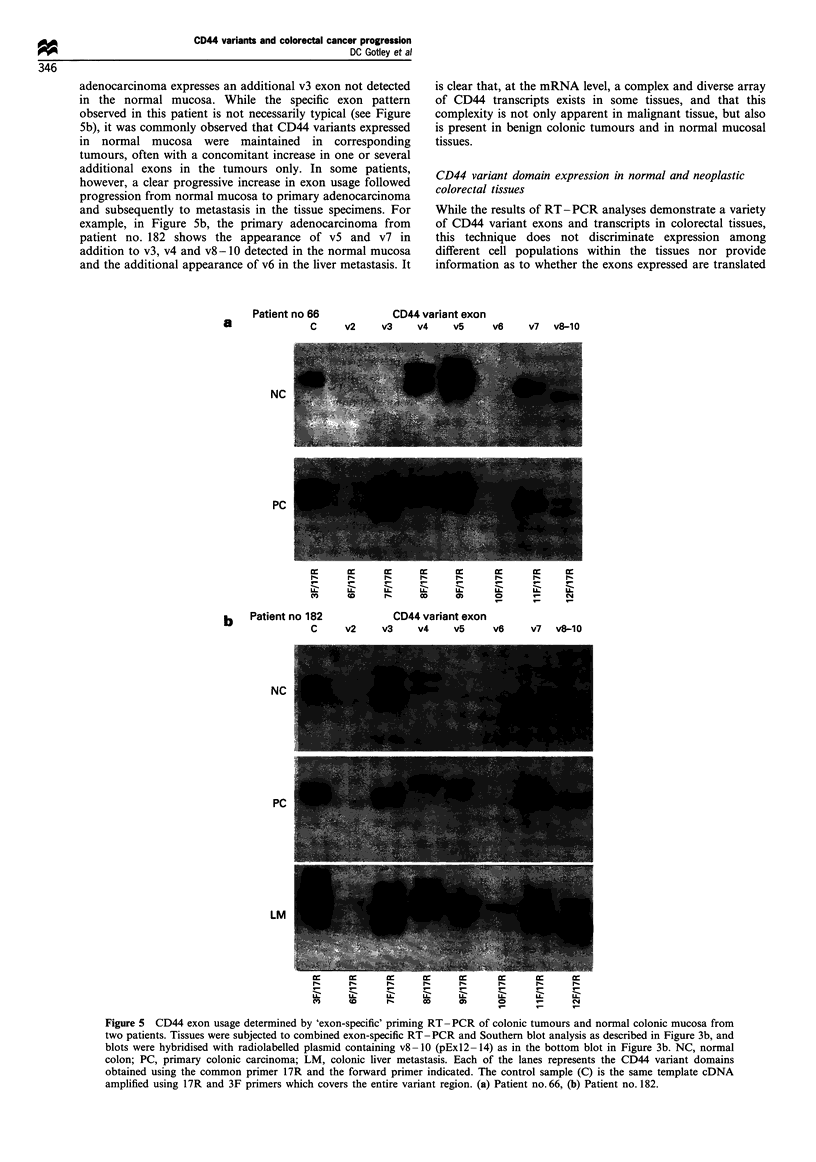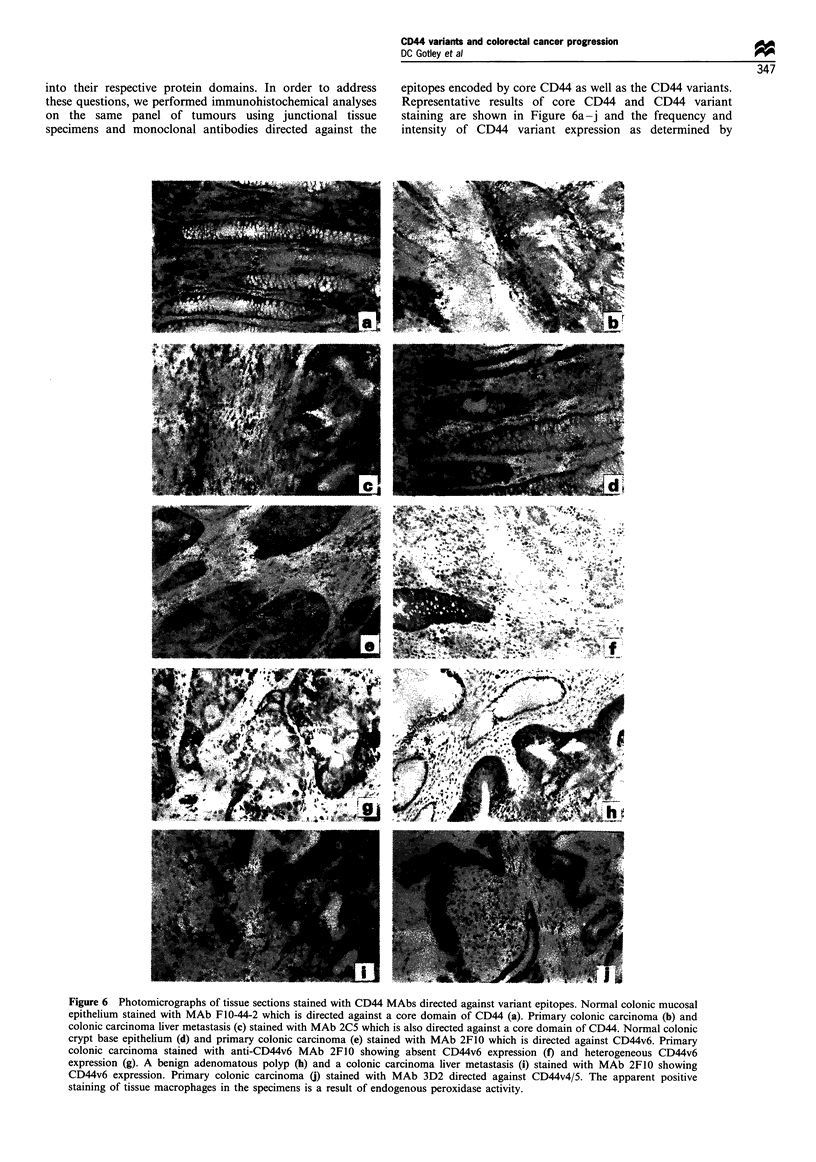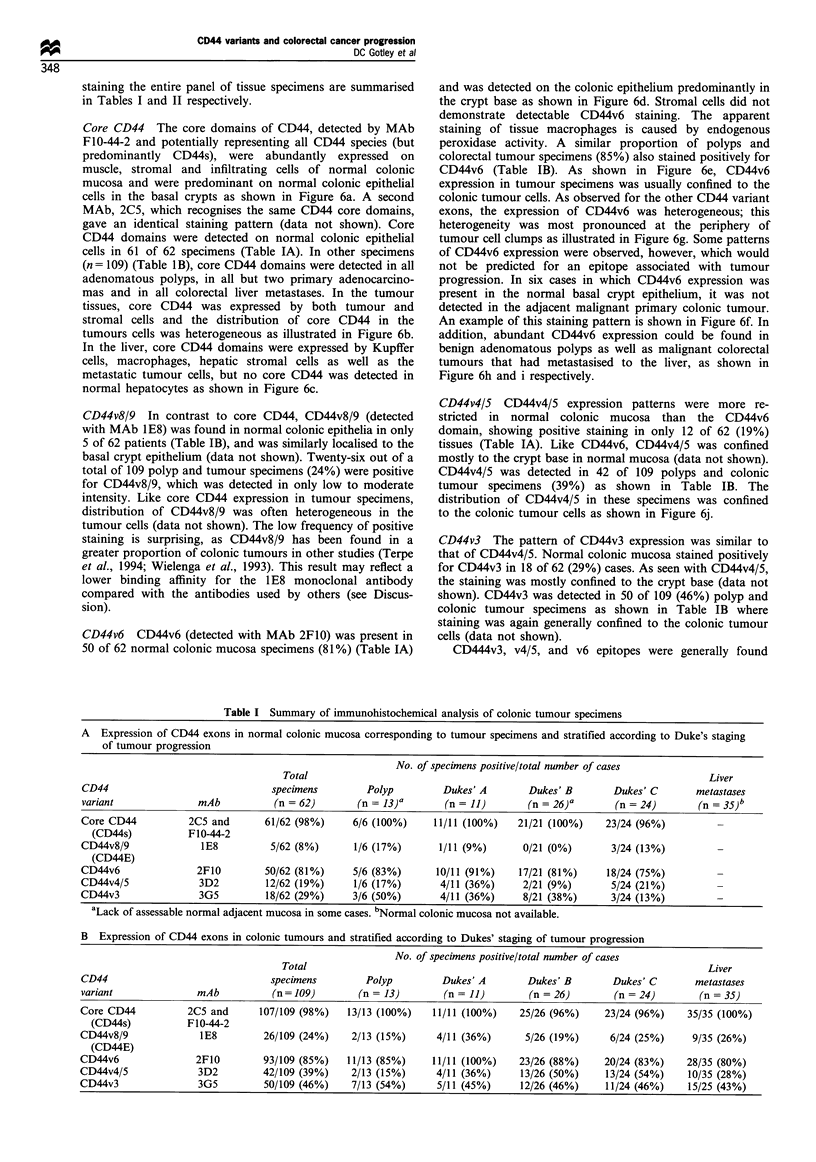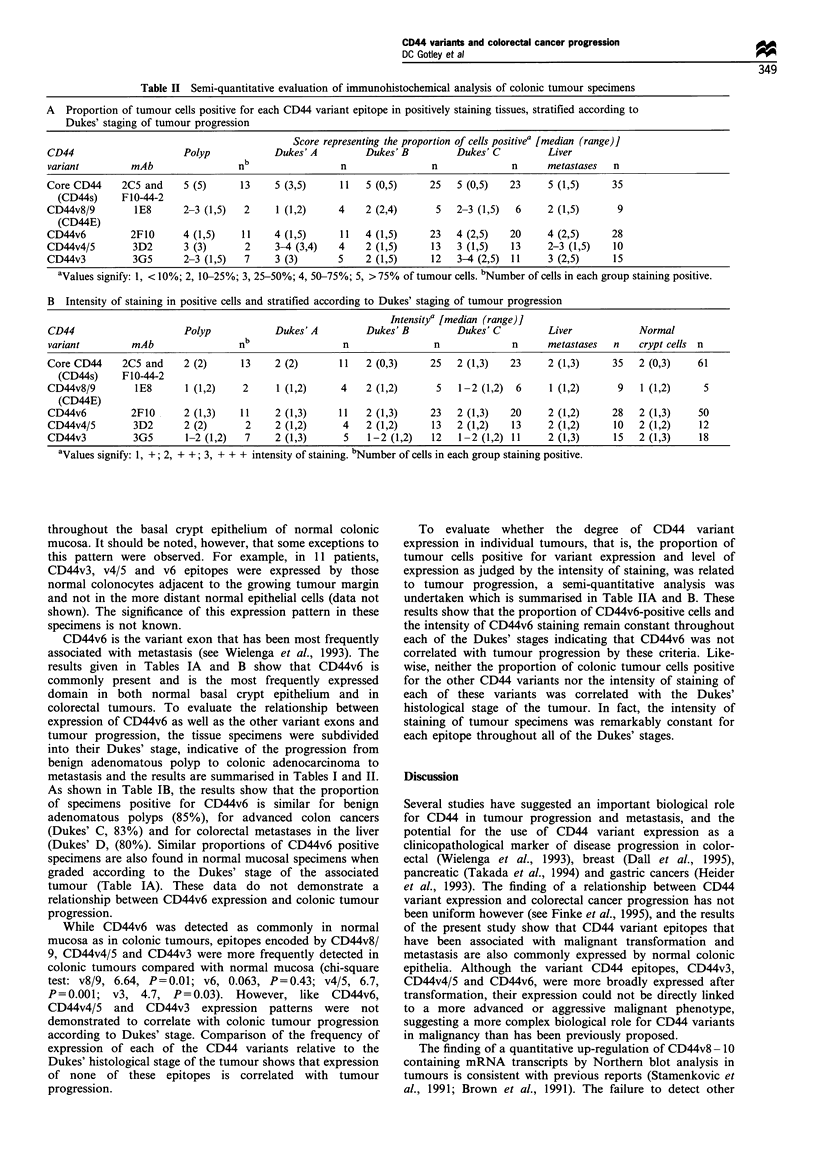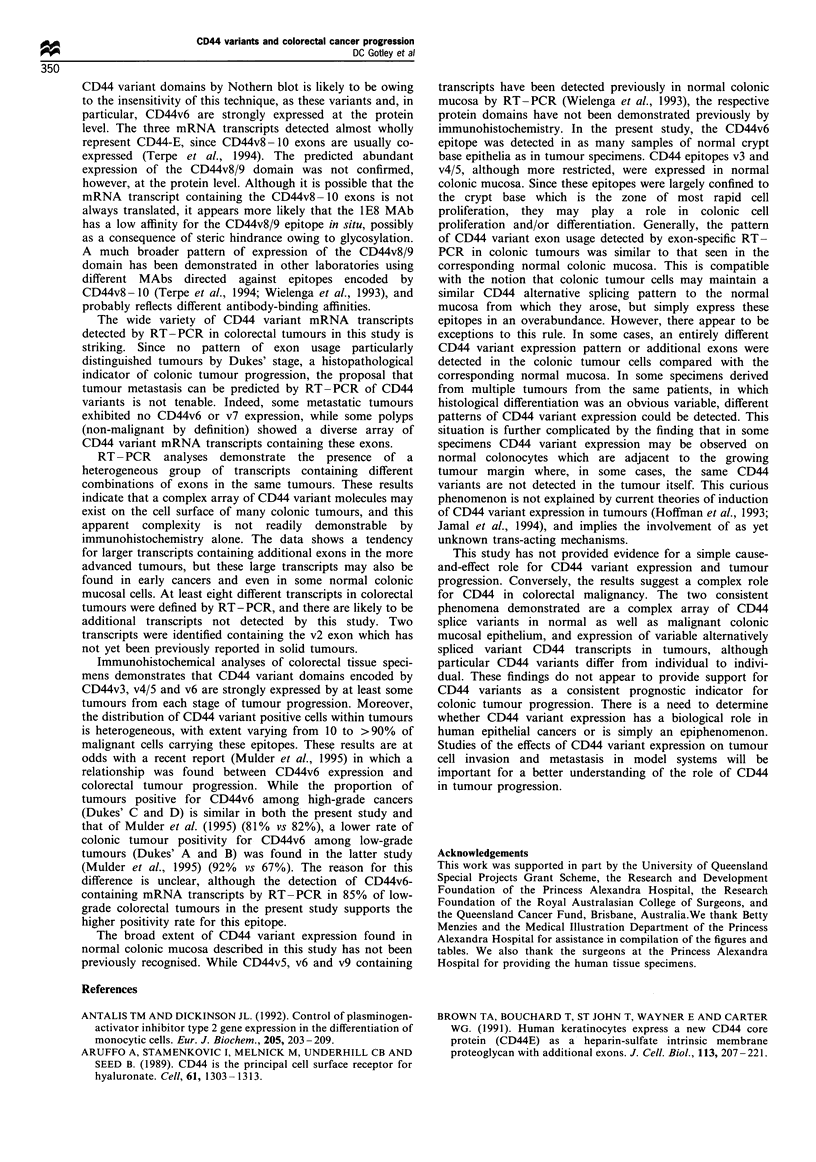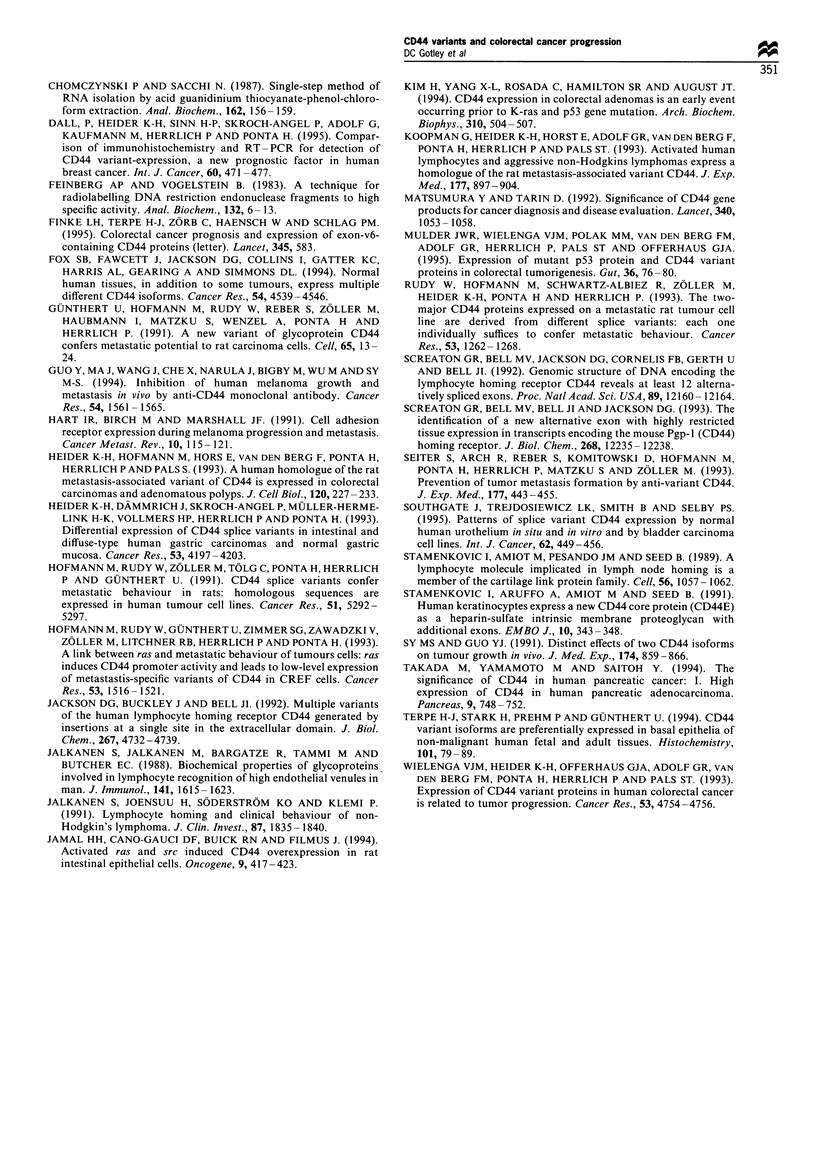# Alternatively spliced variants of the cell adhesion molecule CD44 and tumour progression in colorectal cancer.

**DOI:** 10.1038/bjc.1996.364

**Published:** 1996-08

**Authors:** D. C. Gotley, J. Fawcett, M. D. Walsh, J. A. Reeder, D. L. Simmons, T. M. Antalis

**Affiliations:** Queensland Cancer Fund Experimental Oncology Unit, Queensland Institute of Medical Research, Brisbane, Australia.

## Abstract

**Images:**


British Journal of Cancer (1996) 74, 342-351
© 1996 Stockton Press All rights reserved 0007-0920/96 $12.00

### Alternatively spliced variants of the cell adhesion molecule CD44 and tumour progression in colorectal cancer

DC Gotley[1,2], J Fawcett[3], MD Walsh[2], JA Reeder[1], DL Simmons[3] and TM Antalis[1,4]

[1]*The Queensland Cancer Fund Experimental Oncology Unit, The Queensland Institute of Medical Research;* [2]*Department of Surgery, Princess Alexandra Hospital and Royal Brisbane Hospital, University of Queensland, Brisbane 4029, Australia;* [3]*Cell Adhesion Laboratory and Molecular Oncology Unit, Imperial Cancer Research Fund, Institute of Molecular Medicine, John Radcliffe Hospital, Headington, Oxford OX3 9DU, UK.*

**Summary** Increased expression of alternatively spliced variants of the CD44 family of cell adhesion molecules has been associated with tumour metastasis. In the present study, expression of alternatively spliced variants of CD44 and their cellular distribution have been investigated in human colonic tumours and in the corresponding normal mucosa, in addition to benign adenomatous polyps. The expression of CD44 alternatively spliced variants has been correlated with tumour progression according to Dukes' histological stage. CD44 variant expression was determined by immunohistochemisty using monoclonal antibodies directed against specific CD44 variant domains together with RT-PCR analysis of CD44 variant mRNA expression in the same tissue specimens. We demonstrate that as well as being expressed in colonic tumour cells, the full range of CD44 variants, CD44v2–v10, are widely expressed in normal colonic crypt epithelium, predominantly in the crypt base. CD44v6, the epitope which is most commonly associated with tumour progression and metastasis, was not only expressed by many benign colonic tumours, but was expressed as frequently in normal basal crypt epithelium as in malignant colonic tumour cells, and surprisingly, was even absent from some metastatic colorectal tumours. Expression of none of the CD44 variant epitopes was found to be positively correlated with tumour progression or with colorectal tumour metastasis to the liver, results which are inconsistent with a role for CD44 variants as indicators of colonic cancer progression.

**Keywords:** CD44; colorectal cancer; alternative splicing; tumour progression; metastasis

CD44 is a widely distributed cell adhesion molecule that has been implicated in the metastasis of epithelial tumours (Günthert *et al.*, 1991; Heider *et al.*, 1993) and lymphomas (Jalkanen *et al.*, 1991; Sy and Guo, 1991). There are multiple CD44 isoforms generated by alternative splicing of up to 12 exons, leading to the expansion of a basic 'haematopoietic' transcript (CD44s) into a large family of molecules with potentially diverse functions (Screaton *et al.*, 1992). CD44s is a major cell-surface receptor for hyaluronate and mediates cell-extracellular matrix (ECM) adhesion (Aruffo *et al.*, 1989), and also lymphocyte trafficking via the lymph node high endothelium (Jalkanen *et al.*, 1988).

A range of CD44 alternatively spliced variants, the functions of which are unknown, are expressed in low abundance on some cells of epithelial origin (Terpe *et al.*, 1994), and are also transiently expressed by activated lymphocytes together with CD44s (Koopman *et al.*, 1993). A link between CD44 expression, tumour progression and metastasis has been suggested by studies demonstrating an up-regulation of CD44s and CD44 variants in certain tumours (Jalkanen *et al.*, 1991; Stamenkovic *et al.*, 1989; Wielenga *et al.*, 1993; Hart *et al.*, 1991). The most compelling evidence, however, has come from studies employing animal models, in which transfection of rat CD44 cDNAs containing v6 (exon 10) and v7 (exon 11) into non-metastasising cell lines results in creation of a mestastatic phenotype (Günthert *et al.*, 1991; Rudy *et al.*, 1993). Metastasis in these models can be inhibited by co-treatment with monoclonal antibodies directed against CD44s (Guo *et al.*, 1994), or CD44 variant domains (Seiter *et al.*, 1993). Isoforms of CD44 have been identified in human tumours by either reverse transcription polymerase chain reaction (RT-PCR) or immunohistochemistry (Heider *et al.*, 1993; Jackson *et al.*, 1992; Hofmann *et al.*, 1991), and the results suggest that a disruption in the control of the splicing mechanism may occur in transformed cells. The appearance of splice variants of CD44 occurs soon after neoplastic transformation (Kim *et al.*, 1994), and the expression of the CD44v6 epitope, encoded by exon 10, has been reported to correlate with tumour progression to more advanced stages in colorectal cancer (Wielenga *et al.*, 1993; Mulder *et al.*, 1995).

The observed expression of restricted epitope domains of CD44 in metastatic tumours is important because it provides the potential for both highly specific screening of tissue biopsies for metastatic potential (Matsumura *et al.*, 1992) and for therapeutic targeting of mestastatic disease. However, recent reports documenting expression of CD44v6 domain in a few normal colonic tissues (Terpe *et al.*, 1994; Fox *et al.*, 1994) and in bladder cancer (Southgate *et al.*, 1995) question the notion that expression of CD44 variants is indicative of mestastatic disease and may imply a more complex biological role for CD44 variants in normal cells as well as in neoplastic cells.

In order to clarify the impact of expression of alternatively spliced CD44 variants on colonic cancer progression, we have undertaken a comprehensive study of CD44 variant expression in a large sample of patients and compared the malignant tumour specimens with corresponding normal colonic mucosa from the same patients. We have included benign adenomatous polyps as as well as tissues representative of the range of colorectal tumour progression through to metastasis. As the best marker for colorectal tumour progression remains the histological stage, we have correlated CD44 variant epitope expression with Dukes' histological stage of the tumour. Reverse transcriptase-polymerase chain reaction (RT-PCR) and exon-specific priming have been employed to identify specific mRNA transcripts expressed and the cellular localisation of the respective CD44 variant domains was studied by immunohistochemistry. Analysis of the expression of each of the CD44 alternatively spliced exons, the epitopes encoded by them and their cellular distribution should provide insight into the significance of CD44 variant expression in colorectal cancer.

Correspondence: TM Antalis, The Queensland Institute of Medical Research, Post Office Royal Brisbane Hospital, Queensland 4029, Australia
Received 24 November 1995; revised 13 February 1996; accepted 15 February 1996

### Materials and methods

*Patients and tumour specimens*

A total of 109 patients with colonic neoplasms (adenomatous polyps, carcinomas or liver metastases) underwent colonscopic or surgical resection. Samples of tumour and normal mucosa (at least 20 cm from the tumour) were obtained from surgical specimens and portions were either processed for routine histological examination or snap frozen in liquid nitrogen for RNA analysis. Specimens of junctional epithelium (between tumour and normal mucosa) were also collected for immunohistochemical analyses (see below). Tumours were staged pathologically according to a modified Dukes' system where stage A is tumour confined to the bowel wall, stage B is invasion through the wall, stage C is lymph node involvement and stage D is the presence of distant metastases.

*Northern blot analyses*

Total RNA was obtained from frozen tissue specimens by the acid/guanidinium thiocyanate method of Chomczynski and Sacchi (1987). RNA samples (10 $\mu$g) were electrophoresed on 1.2% denaturing agarose gels containing 1.1% formaldehyde and blotted by capillary diffusion onto Hybond N nylon membranes (Amersham, Castle Hill, Australia). The membranes were hybridised essentially as described (Antalis and Dickinson, 1992) and washed to a final stringency of $0.1 \times$SSC, 0.1% sodium dodecyl sulphate (SDS) at 65°C. Probes directed against specific CD44 variant exons were generated by RT–PCR using total RNA isolated from either the breast cancer cell line ZR75 or a colorectal hepatic metastasis as templates. The amplified DNA was cloned into pGEM-T (Promega, Madison, WI, USA) or pBluescript (Stratagene, La Jolla, CA, USA) and characterised by DNA sequence analysis. Seven CD44 variant probes were obtained and designated pEx6, pEx7, pEx8, pEx9, pEx10, pEx11, pEx12–14 (Figure 1a). The CD44 variant plasmid constructs were radiolabelled by the random priming method for Nothern blot analyses (Feinberg *et al.*, 1983). Specific CD44 sense and antisense riboprobes were transcribed *in vitro* in the presence of [$^{32}$P]UTP using SP6, $T_7$ or $T_3$ promoters as appropriate. Blots were reprobed with a radiolabelled oligonucleotide against human 18S rRNA as a measure of total RNA loaded in each lane (Antalis and Dickinson, 1992). The blots were exposed to Kodak XK-1 film between Dupont Cronex intensifying screens at −70°C for varying times and quantitated by scanning different exposures of autoradiographs using a scanning densitometer (Molecular Dynamics) driven by ImageQuant software.

*CD44 variant-specific reverse transcription polymerase chain reaction (RT–PCR)*

cDNA was synthesised from 5 $\mu$g of total RNA using AMV reverse transcriptase (Promega) in the presence of oligo dT(16–18) (Pharmacia Biotech, Uppsala, Sweden) in a 25 $\mu$l reaction volume at 42°C for 60 min. In each experiment, 1 $\mu$l of this reaction mixture was included in a 50 $\mu$l PCR reaction in the presence of 10 mM Tris HCl pH 8.3, 50 mM potassium chloride, 2 mM magnesium chloride and 0.2 mM dNTP. The PCR amplification conditions were: denaturation at 94°C for 2 min, annealing at 45°C for 1 min and extension at 72°C for 2 min for four cycles; then denaturation at 94°C for 30 s, annealing at 55°C for 30 s and extension at 72°C for 2 min for 28 cycles, with a final extension time of 7 min.

*Detection and cloning of CD44 variant inserts*

To determine the DNA sequences of the CD44 variants expressed in the tissue specimens, variant inserts were isolated by PCR amplification using primers which flank the proximal membrane variant region: the forward primer derived from the 5′ sequence of exon 3, 3F (5′-TCCCAGTATGACACATATTGC-3′) and a reverse primer derived from exon 17, 17R (5′-CCAAGATGATCAGCCATTCTGG-3′) (Hoffman *et al.*, 1991) (Figure 1a). PCR products were separated by agarose gel electrophoresis, purified and ligated into pGEM-T (Promega). Transformants containing CD44 variant inserts were identified and characterised by DNA sequence analysis.

*Detection of variant domains by exon-specific priming*

A technique of 'exon-specific' priming was validated and used to determine CD44 variant expression patterns in the tissue specimens. In separate reactions, forward primers derived from the 5′ sequences of exon 3 (3F, described above), 6F (v2), 7F (v3), 8F (v4), 9F (v5), 10F (v6), 11F (v7) and 12F (v8) were each used with a common reverse primer derived from exon 17 (17R, as above). The positions of the CD44 variant primers are shown in Figure 1a and the sequences were as follows:

v2: 5′-GATGAGCACTAGTGCTACAGCAAC-3′
v3: 5′-GTACGTCTTCAAATACCATCTCAG-3′
v4: 5′-CAACCACACCACGGGCTTTTGACC-3′
v5: 5′-ATGTAGACAGAAATGGCACCACTG-3′
v6: 5′-TCCAGGCAACTCCTAGTAGTACAA-3′
v7: 5′-CAGCCTCAGCTCATACCAGCCATC-3′
v8: 5′-ATATGGACTCCAGTCATAGTACAA-3′

RNA isolated from each tissue specimen was reverse transcribed with AMV reverse transcriptase. The cDNA was used as the template for eight PCR reactions (1 $\mu$l in each reaction), each containing the common reverse primer 17R and one of the forward primers above. The same cDNA was used for the control reactions using primers 3F and 17R which spans the entire variant region and controls for the integrity of the mRNA and the reproducibility of the RT–PCR process itself. Each PCR reaction mixture (10 $\mu$l per lane) was resolved on a non-denaturing 1.2% agarose gel and

**Figure 1** (a) Schematic representation of the genomic structure of the CD44 gene. Constitutively expressed exons, ▭; exons capable of alternative splicing, ▩. Domains to which CD44 variant DNA probes, CD44 monoclonal antibodies and oligonucleotide primers used in RT–PCR analyses are directed are shown. (b) CD44 variant exons amplified by RT–PCR with oligonucleotide primers 3F and 17R using RNA from four pooled colonic tumours. Amplified products were cloned and characterised by Southern blotting and DNA sequence analyses. The CD44 variant exons detected, ▤.

Southern blotted by capillary transfer to a Hybond N nylon membrane followed by hybridisation with radiolabelled probes as described above.

*Immunohistochemistry*

Monoclonal antibody (MAb) F10-44-2 recognises a 'core' domain of CD44s and was obtained from Serotec (Oxford, UK). Other MAbs, 2C5 (subclass $IgG_{2a}$, anti-CD44s), 3G5 (subclass $IgG_{2b}$, anti-v3), 3D2 (subclass $IgG_1$, anti-v4/5) 2F10 (subclass $IgG_1$, anti-v6), 2G9 (subclass IgM, anti-v6) and 1E8 (subclass IgM, anti-v8/9) were produced as described (Fox *et al.*, 1994). Essentially mice were immunised with purified soluble recombinant proteins generated by transfection into COS1 cells of molecular chimaeras comprising CD44 variant exon sequences fused to a CD33 signal sequence and an $IgG_1$-Fc sequence in pCDM8 (Invitrogen, The Netherlands). The specificities of the MAbs obtained were determined by immunostaining of specific CD44 variant constructs transfected into COS1 cells.

For immunohistochemical analyses of tissues, specimens were embedded in OCT compound (Miles Laboratories, Elkhart, IN, USA), quenched in liquid nitrogen and stored at −70°C until sectioned. Frozen sections (5 μm) were affixed to Vectabond-treated slides (Vector Laboratories, Burlingame, CA, USA) and air dried before fixation in equal parts chloroform/acetone. After rehydration in phosphate-buffered saline (PBS), endogenous peroxidase activity was inhibited with 0.3% hydrogen peroxide and 0.1% sodium azide, and non-specific antibody binding was inhibited by immersion in 4% non-fat skimmed milk powder. Endogenous biotin activity was blocked with 0.1% avidin (Dakopatts, Carpinteria, CA, USA) followed by 0.01% D-biotin. Non-specific antibody binding was further inhibited by preincubating sections with 10% non-immune goat serum. Sections were incubated with primary antibody (or no antibody for control) for 30 min (RT), then biotinylated goat anti-mouse immunoglobulins (Zymed, San Francisco, CA, USA) followed by streptavidin–horseradish peroxidase complex (Zymed). Antigenic sites were demonstrated using 3,3′-diaminobenzidine as chromogen with hydrogen peroxide as substrate. Sections were counterstained with Harris' haematoxylin and mounted in Permount.

CD44 expression was assessed with regard to staining intensity (0, 1+, 2+ or 3+), proportion of positive cells (<10%, 10–25%, 25–50%, 50–75% or >75%) and localisation (basal cells, full thickness or scattered cells).

*Statistical analyses*

Comparative data were analysed using the Wilcoxon rank sum test.

### Results

*Northern blot analysis detects CD44v8–10 expression but not CD44v3–7*

A total of 37 colon tumours (polyps, $n=6$; Dukes' stages A, $n=8$; B, $n=10$ and C, $n=13$) together with matched normal colonic mucosa and 17 liver metastases were examined by Northern blot analysis for expression of CD44 variants using radiolabelled probes directed against exons v3, v6, v7 and v8–10. Three CD44 mRNA transcripts containing v8–10 (2.0 kb, 2.6 kb and 5.6 kb) were detected in all colonic neoplasms including adenomatous polyps and liver metastases. Representative blots are shown in Figure 2a and b. These transcripts were not detected, however, in any specimen of normal colonic mucosa (Figure 2a) nor in normal liver tissues (Figure 2b) by Northern blot analysis. Northern blots of the same tissue specimens hybridised with variant-specific probes for v3, v6, and v7 resulted in detection of no CD44 transcripts containing these variant exons, even when the membranes were probed using sensitive *in vitro* generated riboprobes. This may be due to the insensitivity of the Northern blot technique in detecting low abundance mRNA transcripts, which may also be compounded by the use of shorter exon-specific probes in comparison with the larger CD44v8–10 probe.

**Figure 2** Northern blot analysis of (**a**) normal colon (N) and primary colonic tumours (P), and (**b**) normal liver (N) and colorectal liver metastases (LM) using an antisense radiolabelled riboprobe directed against the CD44v8–10 domain (CD44-E). As a control for the amount of RNA loaded in each lane, each blot was reprobed using a radiolabelled oligonucleotide directed against 18S ribsomal RNA.

*Multiple CD44 variant transcripts are detected by reverse transcription–polymerase chain reaction (RT–PCR)*

In order to detect the presence of low abundance alternatively spliced CD44 mRNA transcripts, RT–PCR was employed using primers flanking the proximal membrane extracellular domain, 3F and 17R (see Figure 1a). Multiple amplified products representing different CD44 variant transcripts were detected in all specimens of normal colonic mucosa and tumours analysed from 57 patients. A representative sample is shown in Figure 3a. Both normal and neoplastic colon tissues expressed both the standard (CD44s) (data not shown) and epithelial (CD44E, CD44v8–10) transcripts. In addition, these tisuses expressed slower migrating isoforms of CD44 as shown in Figure 3a.

In order to examine the range of CD44 variant exons expressed in the slower migrating isoforms, a technique termed 'exon-specific' RT–PCR was used. cDNA was amplified by PCR using exon-specific forward primers together with a common reverse primer. 17R, Southern blotted and hybridised with radiolabelled probes specific for each CD44 variant exon. Figure 3 shows an example of the application of this technique to a colorectal liver metastasis which had demonstrated a range of slower migrating isoforms of CD44. Analysis of this tissue specimen by exon-specific RT–PCR and Southern blotting demonstrated that this tumour specimen expressed the full range of alternatively spliced CD44 variant exons (v2–v10).

In order to investigate CD44 variant exon usage in randomly selected, individual CD44 mRNA transcripts contained within the tumour tissues, cDNA synthesised from four different colonic tumour specimens was pooled, amplified by PCR using primers flanking the proximal membrane extracellular domain, and the amplified products cloned into pGEM-T and sequenced. Eight different CD44 variant clones representing variant transcripts each displaying a different pattern of exon usage were obtained as shown in Figure 1b. The majority of the CD44 variant transcripts identified by this technique (six out of eight) displayed a sequential pattern of variant exon expression; i.e. if the first variant exon used was v5, then v6, v7, v8, v9 and v10 were also incorporated into the transcript. In two variants, however, a discontinuous variant exon expression pattern was obtained [v3, v5–v10 (no. 7) and v2–3, v8–10 (no. 8)] and one of the transcripts was found to incorporate the full range of variant exons, v2–10 (no. 6).

**Figure 3** Southern blot analysis of CD44 variants amplified by RT–PCR. (**a**) cDNA obtained from RNA isolated from normal colon (N), primary colonic tumours (P), normal liver (NL) and colorectal liver metastases (ML) was amplified using primers flanking the proximal membrane extracellular domains, 3F and 17R. Amplification products were separated on 1.2% agarose gels, transferred to nylon membrane and probed with radiolabelled pEx12–14, a plasmid containing the CD44 variant region of CD44E. (**b**) The 'exon-specific' priming technique to detect specific CD44 variant exon usage in colorectal tissues. cDNA derived from a colorectal liver metastasis containing the full range of CD44 variant exons was amplified using forward primers 6F, 7F, 8F, 9F, 10F, 11F, 12–14F respectively and the reverse primer 17R. The amplification products were analysed on replicate 1.2% agarose gels and each gel blotted onto a Hybond N nylon membrane. The seven identical blots obtained were each hybridised with a different radiolabelled plasmid containing the CD44 variant exons v2 (pEx6), v3 (pEx7), v4 (pEx8), v5 (pEx9), v7 (pEx11), or v8–10 (pEx 12–14) to show the specific variant domains and the utility of the technique. Thus, analysis of a template of unknown CD44 variant composition, using the conditions in the bottom blot (pEx12–14 to probe the panel of PCR reactions each primed with a different forward primer) will give a comprehensive profile of the specific CD44 variant exons present in that specimen, i.e. a positive signal in lane 1 indicating the presence of exon 6 (v2), lane 2 the presence of exon 7 (v3), etc.

*Normal and neoplastic colon tissues express a wide range of CD44 variant exons*

To evaluate CD44 variant exon expression in colonic tumours and to attempt to correlate specific variant exons with tumour progression, 'exon-specific' RT–PCR was applied to a panel of colonic tumour tissues representative of each stage of tumour progression according to Dukes' stage, as well as benign adenomatous polyps. The presence of each variant was scored and the percentage of tumours expressing each CD44 variant exon was compared according to Dukes' stage as shown in Figure 4. The data show that no individual CD44 variant exon was clearly associated with colorectal tumour progression and each variant exon from v2 through to v8–v10 was detected in tumours from each Dukes' stage. Expression of CD44v1 was not detected in any tissue specimen. Although v1 is expressed in the mouse, it has not been found in human CD44 alternatively spliced transcripts (Screaton *et al.*, 1993). CD44v2-containing transcripts were detected in several tumour specimens; the expression of this domain has not been reported previously in human epithelial tumours. Several combinations of CD44 exon variants were detected in adenomatous polyps and Dukes' A, B and C tumours, but transcripts containing all of the CD44 variant exons (v2–v10) were found only in colorectal metastases in the liver (Dukes' D).

As observed for the colonic tumour specimens, normal colonic mucosa also demonstrated similar heterogenity in the patterns of CD44 variant exon usage and there was no consistent pattern in the variant exon usage with progression to malignancy (data not shown). Examples of 'exon-specific' RT–PCR performed on normal mucosa from two patients compared with their malignant counterparts are shown in Figure 5. In Figure 5a, the normal colonic mucosa expresses CD44 variant exons v4, v5, v7 and v8–v10 in common with the corresponding colonic adenocarcinoma, and the colonic

**Figure 4** The pattern of CD44 variant exon usage in colorectal tumours grouped according to Dukes' histological staging. Exon-specific RT–PCR was performed on RNA isolated from colonic tumour specimens from 57 patients as described in Materials and methods. Amplification products were analysed by Southern blotting and probed with radiolabelled plasmids containing exon-specific cDNA sequences. The percentage of tumours expressing each CD44 variant exon was determined according to Dukes' stage as indicated. The number of specimens in each group are: polyps (■), $n=6$; Dukes' A (□), $n=8$; Dukes' B (▨), $n=10$; Dukes' C (▩), $n=13$; Dukes' D (▥), $n=17$.

adenocarcinoma expresses an additional v3 exon not detected in the normal mucosa. While the specific exon pattern observed in this patient is not necessarily typical (see Figure 5b), it was commonly observed that CD44 variants expressed in normal mucosa were maintained in corresponding tumours, often with a concomitant increase in one or several additional exons in the tumours only. In some patients, however, a clear progressive increase in exon usage followed progression from normal mucosa to primary adenocarcinoma and subsequently to metastasis in the tissue specimens. For example, in Figure 5b, the primary adenocarcinoma from patient no. 182 shows the appearance of v5 and v7 in addition to v3, v4 and v8–10 detected in the normal mucosa and the additional appearance of v6 in the liver metastasis. It is clear that, at the mRNA level, a complex and diverse array of CD44 transcripts exists in some tissues, and that this complexity is not only apparent in malignant tissue, but also is present in benign colonic tumours and in normal mucosal tissues.

### *CD44 variant domain expression in normal and neoplastic colorectal tissues*

While the results of RT–PCR analyses demonstrate a variety of CD44 variant exons and transcripts in colorectal tissues, this technique does not discriminate expression among different cell populations within the tissues nor provide information as to whether the exons expressed are translated

**Figure 5** CD44 exon usage determined by 'exon-specific' priming RT–PCR of colonic tumours and normal colonic mucosa from two patients. Tissues were subjected to combined exon-specific RT–PCR and Southern blot analysis as described in Figure 3b, and blots were hybridised with radiolabelled plasmid containing v8–10 (pEx12–14) as in the bottom blot in Figure 3b. NC, normal colon; PC, primary colonic carcinoma; LM, colonic liver metastasis. Each of the lanes represents the CD44 variant domains obtained using the common primer 17R and the forward primer indicated. The control sample (C) is the same template cDNA amplified using 17R and 3F primers which covers the entire variant region. (**a**) Patient no. 66, (**b**) Patient no. 182.

into their respective protein domains. In order to address these questions, we performed immunohistochemical analyses on the same panel of tumours using junctional tissue specimens and monoclonal antibodies directed against the epitopes encoded by core CD44 as well as the CD44 variants. Representative results of core CD44 and CD44 variant staining are shown in Figure 6a–j and the frequency and intensity of CD44 variant expression as determined by

**Figure 6** Photomicrographs of tissue sections stained with CD44 MAbs directed against variant epitopes. Normal colonic mucosal epithelium stained with MAb F10-44-2 which is directed against a core domain of CD44 (**a**). Primary colonic carcinoma (**b**) and colonic carcinoma liver metastasis (**c**) stained with MAb 2C5 which is also directed against a core domain of CD44. Normal colonic crypt base epithelium (**d**) and primary colonic carcinoma (**e**) stained with MAb 2F10 which is directed against CD44v6. Primary colonic carcinoma stained with anti-CD44v6 MAb 2F10 showing absent CD44v6 expression (**f**) and heterogeneous CD44v6 expression (**g**). A benign adenomatous polyp (**h**) and a colonic carcinoma liver metastasis (**i**) stained with MAb 2F10 showing CD44v6 expression. Primary colonic carcinoma (**j**) stained with MAb 3D2 directed against CD44v4/5. The apparent positive staining of tissue macrophages in the specimens is a result of endogenous peroxidase activity.

staining the entire panel of tissue specimens are summarised in Tables I and II respectively.

*Core CD44* The core domains of CD44, detected by MAb F10-44-2 and potentially representing all CD44 species (but predominantly CD44s), were abundantly expressed on muscle, stromal and infiltrating cells of normal colonic mucosa and were predominant on normal colonic epithelial cells in the basal crypts as shown in Figure 6a. A second MAb, 2C5, which recognises the same CD44 core domains, gave an identical staining pattern (data not shown). Core CD44 domains were detected on normal colonic epithelial cells in 61 of 62 specimens (Table IA). In other specimens ($n=109$) (Table 1B), core CD44 domains were detected in all adenomatous polyps, in all but two primary adenocarcinomas and in all colorectal liver metastases. In the tumour tissues, core CD44 was expressed by both tumour and stromal cells and the distribution of core CD44 in the tumours cells was heterogeneous as illustrated in Figure 6b. In the liver, core CD44 domains were expressed by Kupffer cells, macrophages, hepatic stromal cells as well as the metastatic tumour cells, but no core CD44 was detected in normal hepatocytes as shown in Figure 6c.

*CD44v8/9* In contrast to core CD44, CD44v8/9 (detected with MAb 1E8) was found in normal colonic epithelia in only 5 of 62 patients (Table IB), and was similarly localised to the basal crypt epithelium (data not shown). Twenty-six out of a total of 109 polyp and tumour specimens (24%) were positive for CD44v8/9, which was detected in only low to moderate intensity. Like core CD44 expression in tumour specimens, distribution of CD44v8/9 was often heterogeneous in the tumour cells (data not shown). The low frequency of positive staining is surprising, as CD44v8/9 has been found in a greater proportion of colonic tumours in other studies (Terpe *et al.*, 1994; Wielenga *et al.*, 1993). This result may reflect a lower binding affinity for the 1E8 monoclonal antibody compared with the antibodies used by others (see Discussion).

*CD44v6* CD44v6 (detected with MAb 2F10) was present in 50 of 62 normal colonic mucosa specimens (81%) (Table IA) and was detected on the colonic epithelium predominantly in the crypt base as shown in Figure 6d. Stromal cells did not demonstrate detectable CD44v6 staining. The apparent staining of tissue macrophages is caused by endogenous peroxidase activity. A similar proportion of polyps and colorectal tumour specimens (85%) also stained positively for CD44v6 (Table IB). As shown in Figure 6e, CD44v6 expression in tumour specimens was usually confined to the colonic tumour cells. As observed for the other CD44 variant exons, the expression of CD44v6 was heterogeneous; this heterogeneity was most pronounced at the periphery of tumour cell clumps as illustrated in Figure 6g. Some patterns of CD44v6 expression were observed, however, which would not be predicted for an epitope associated with tumour progression. In six cases in which CD44v6 expression was present in the normal basal crypt epithelium, it was not detected in the adjacent malignant primary colonic tumour. An example of this staining pattern is shown in Figure 6f. In addition, abundant CD44v6 expression could be found in benign adenomatous polyps as well as malignant colorectal tumours that had metastasised to the liver, as shown in Figure 6h and i respectively.

*CD44v4/5* CD44v4/5 expression patterns were more restricted in normal colonic mucosa than the CD44v6 domain, showing positive staining in only 12 of 62 (19%) tissues (Table IA). Like CD44v6, CD44v4/5 was confined mostly to the crypt base in normal mucosa (data not shown). CD44v4/5 was detected in 42 of 109 polyps and colonic tumour specimens (39%) as shown in Table IB. The distribution of CD44v4/5 in these specimens was confined to the colonic tumour cells as shown in Figure 6j.

*CD44v3* The pattern of CD44v3 expression was similar to that of CD44v4/5. Normal colonic mucosa stained positively for CD44v3 in 18 of 62 (29%) cases. As seen with CD44v4/5, the staining was mostly confined to the crypt base (data not shown). CD44v3 was detected in 50 of 109 (46%) polyp and colonic tumour specimens as shown in Table IB where staining was again generally confined to the colonic tumour cells (data not shown).

CD444v3, v4/5, and v6 epitopes were generally found

**Table I** Summary of immunohistochemical analysis of colonic tumour specimens

A Expression of CD44 exons in normal colonic mucosa corresponding to tumour specimens and stratified according to Duke's staging of tumour progression

| *CD44 variant* | *mAb* | *Total specimens (n = 62)* | *Polyp (n = 13)[a]* | *Dukes' A (n = 11)* | *Dukes' B (n = 26)[a]* | *Dukes' C (n = 24)* | *Liver metastases (n = 35)[b]* |
|---|---|---|---|---|---|---|---|
| | | | *No. of specimens positive/total number of cases* | | | | |
| Core CD44 (CD44s) | 2C5 and F10-44-2 | 61/62 (98%) | 6/6 (100%) | 11/11 (100%) | 21/21 (100%) | 23/24 (96%) | – |
| CD44v8/9 (CD44E) | 1E8 | 5/62 (8%) | 1/6 (17%) | 1/11 (9%) | 0/21 (0%) | 3/24 (13%) | – |
| CD44v6 | 2F10 | 50/62 (81%) | 5/6 (83%) | 10/11 (91%) | 17/21 (81%) | 18/24 (75%) | – |
| CD44v4/5 | 3D2 | 12/62 (19%) | 1/6 (17%) | 4/11 (36%) | 2/21 (9%) | 5/24 (21%) | – |
| CD44v3 | 3G5 | 18/62 (29%) | 3/6 (50%) | 4/11 (36%) | 8/21 (38%) | 3/24 (13%) | – |

[a]Lack of assessable normal adjacent mucosa in some cases. [b]Normal colonic mucosa not available.

B Expression of CD44 exons in colonic tumours and stratified according to Dukes' staging of tumour progression

| *CD44 variant* | *mAb* | *Total specimens (n=109)* | *Polyp (n = 13)* | *Dukes' A (n = 11)* | *Dukes' B (n = 26)* | *Dukes' C (n = 24)* | *Liver metastases (n = 35)* |
|---|---|---|---|---|---|---|---|
| | | | *No. of specimens positive/total number of cases* | | | | |
| Core CD44 (CD44s) | 2C5 and F10-44-2 | 107/109 (98%) | 13/13 (100%) | 11/11 (100%) | 25/26 (96%) | 23/24 (96%) | 35/35 (100%) |
| CD44v8/9 (CD44E) | 1E8 | 26/109 (24%) | 2/13 (15%) | 4/11 (36%) | 5/26 (19%) | 6/24 (25%) | 9/35 (26%) |
| CD44v6 | 2F10 | 93/109 (85%) | 11/13 (85%) | 11/11 (100%) | 23/26 (88%) | 20/24 (83%) | 28/35 (80%) |
| CD44v4/5 | 3D2 | 42/109 (39%) | 2/13 (15%) | 4/11 (36%) | 13/26 (50%) | 13/24 (54%) | 10/35 (28%) |
| CD44v3 | 3G5 | 50/109 (46%) | 7/13 (54%) | 5/11 (45%) | 12/26 (46%) | 11/24 (46%) | 15/25 (43%) |

**Table II** Semi-quantitative evaluation of immunohistochemical analysis of colonic tumour specimens

A Proportion of tumour cells positive for each CD44 variant epitope in positively staining tissues, stratified according to Dukes' staging of tumour progression

| | | *Score representing the proportion of cells positive[a] [median (range)]* | | | | | | | | | |
|---|---|---|---|---|---|---|---|---|---|---|---|
| *CD44 variant* | *mAb* | *Polyp* | n[b] | *Dukes' A* | n | *Dukes' B* | n | *Dukes' C* | n | *Liver metastases* | n |
| Core CD44 (CD44s) | 2C5 and F10-44-2 | 5 (5) | 13 | 5 (3,5) | 11 | 5 (0,5) | 25 | 5 (0,5) | 23 | 5 (1,5) | 35 |
| CD44v8/9 (CD44E) | 1E8 | 2–3 (1,5) | 2 | 1 (1,2) | 4 | 2 (2,4) | 5 | 2–3 (1,5) | 6 | 2 (1,5) | 9 |
| CD44v6 | 2F10 | 4 (1,5) | 11 | 4 (1,5) | 11 | 4 (1,5) | 23 | 4 (2,5) | 20 | 4 (2,5) | 28 |
| CD44v4/5 | 3D2 | 3 (3) | 2 | 3–4 (3,4) | 4 | 2 (1,5) | 13 | 3 (1,5) | 13 | 2–3 (1,5) | 10 |
| CD44v3 | 3G5 | 2–3 (1,5) | 7 | 3 (3) | 5 | 2 (1,5) | 12 | 3–4 (2,5) | 11 | 3 (2,5) | 15 |

[a]Values signify: 1, <10%; 2, 10–25%; 3, 25–50%; 4, 50–75%; 5, >75% of tumour cells. [b]Number of cells in each group staining positive.

B Intensity of staining in positive cells and stratified according to Dukes' staging of tumour progression

| | | *Intensity[a] [median (range)]* | | | | | | | | | | | |
|---|---|---|---|---|---|---|---|---|---|---|---|---|---|
| *CD44 variant* | *mAb* | *Polyp* | n[b] | *Dukes' A* | n | *Dukes' B* | n | *Dukes' C* | n | *Liver metastases* | *n* | *Normal crypt cells* | n |
| Core CD44 (CD44s) | 2C5 and F10-44-2 | 2 (2) | 13 | 2 (2) | 11 | 2 (0,3) | 25 | 2 (1,3) | 23 | 2 (1,3) | 35 | 2 (0,3) | 61 |
| CD44v8/9 (CD44E) | 1E8 | 1 (1,2) | 2 | 1 (1,2) | 4 | 2 (1,2) | 5 | 1–2 (1,2) | 6 | 1 (1,2) | 9 | 1 (1,2) | 5 |
| CD44v6 | 2F10 | 2 (1,3) | 11 | 2 (1,3) | 11 | 2 (1,3) | 23 | 2 (1,3) | 20 | 2 (1,2) | 28 | 2 (1,3) | 50 |
| CD44v4/5 | 3D2 | 2 (2) | 2 | 2 (1,2) | 4 | 2 (1,2) | 13 | 2 (1,2) | 13 | 2 (1,2) | 10 | 2 (1,2) | 12 |
| CD44v3 | 3G5 | 1–2 (1,2) | 7 | 2 (1,3) | 5 | 1–2 (1,2) | 12 | 1–2 (1,2) | 11 | 2 (1,3) | 15 | 2 (1,3) | 18 |

[a]Values signify: 1, +; 2, + +; 3, + + + intensity of staining. [b]Number of cells in each group staining positive.

throughout the basal crypt epithelium of normal colonic mucosa. It should be noted, however, that some exceptions to this pattern were observed. For example, in 11 patients, CD44v3, v4/5 and v6 epitopes were expressed by those normal colonocytes adjacent to the growing tumour margin and not in the more distant normal epithelial cells (data not shown). The significance of this expression pattern in these specimens is not known.

CD44v6 is the variant exon that has been most frequently associated with metastasis (see Wielenga *et al.*, 1993). The results given in Tables IA and B show that CD44v6 is commonly present and is the most frequently expressed domain in both normal basal crypt epithelium and in colorectal tumours. To evaluate the relationship between expression of CD44v6 as well as the other variant exons and tumour progression, the tissue specimens were subdivided into their Dukes' stage, indicative of the progression from benign adenomatous polyp to colonic adenocarcinoma to metastasis and the results are summarised in Tables I and II. As shown in Table IB, the results show that the proportion of specimens positive for CD44v6 is similar for benign adenomatous polyps (85%), for advanced colon cancers (Dukes' C, 83%) and for colorectal metastases in the liver (Dukes' D, (80%). Similar proportions of CD44v6 positive specimens are also found in normal mucosal specimens when graded according to the Dukes' stage of the associated tumour (Table IA). These data do not demonstrate a relationship between CD44v6 expression and colonic tumour progression.

While CD44v6 was detected as commonly in normal mucosa as in colonic tumours, epitopes encoded by CD44v8/9, CD44v4/5 and CD44v3 were more frequently detected in colonic tumours compared with normal mucosa (chi-square test: v8/9, 6.64, $P=0.01$; v6, 0.063, $P=0.43$; v4/5, 6.7, $P=0.001$; v3, 4.7, $P=0.03$). However, like CD44v6, CD44v4/5 and CD44v3 expression patterns were not demonstrated to correlate with colonic tumour progression according to Dukes' stage. Comparison of the frequency of expression of each of the CD44 variants relative to the Dukes' histological stage of the tumour shows that expression of none of these epitopes is correlated with tumour progression.

To evaluate whether the degree of CD44 variant expression in individual tumours, that is, the proportion of tumour cells positive for variant expression and level of expression as judged by the intensity of staining, was related to tumour progression, a semi-quantitative analysis was undertaken which is summarised in Table IIA and B. These results show that the proportion of CD44v6-positive cells and the intensity of CD44v6 staining remain constant throughout each of the Dukes' stages indicating that CD44v6 was not correlated with tumour progression by these criteria. Likewise, neither the proportion of colonic tumour cells positive for the other CD44 variants nor the intensity of staining of each of these variants was correlated with the Dukes' histological stage of the tumour. In fact, the intensity of staining of tumour specimens was remarkably constant for each epitope throughout all of the Dukes' stages.

### Discussion

Several studies have suggested an important biological role for CD44 in tumour progression and metastasis, and the potential for the use of CD44 variant expression as a clinicopathological marker of disease progression in colorectal (Wielenga *et al.*, 1993), breast (Dall *et al.*, 1995), pancreatic (Takada *et al.*, 1994) and gastric cancers (Heider *et al.*, 1993). The finding of a relationship between CD44 variant expression and colorectal cancer progression has not been uniform however (see Finke *et al.*, 1995), and the results of the present study show that CD44 variant epitopes that have been associated with malignant transformation and metastasis are also commonly expressed by normal colonic epithelia. Although the variant CD44 epitopes, CD44v3, CD44v4/5 and CD44v6, were more broadly expressed after transformation, their expression could not be directly linked to a more advanced or aggressive malignant phenotype, suggesting a more complex biological role for CD44 variants in malignancy than has been previously proposed.

The finding of a quantitative up-regulation of CD44v8–10 containing mRNA transcripts by Northern blot analysis in tumours is consistent with previous reports (Stamenkovic *et al.*, 1991; Brown *et al.*, 1991). The failure to detect other

CD44 variant domains by Nothern blot is likely to be owing to the insensitivity of this technique, as these variants and, in particular, CD44v6 are strongly expressed at the protein level. The three mRNA transcripts detected almost wholly represent CD44-E, since CD44v8–10 exons are usually co-expressed (Terpe *et al.*, 1994). The predicted abundant expression of the CD44v8/9 domain was not confirmed, however, at the protein level. Although it is possible that the mRNA transcript containing the CD44v8–10 exons is not always translated, it appears more likely that the 1E8 MAb has a low affinity for the CD44v8/9 epitope *in situ*, possibly as a consequence of steric hindrance owing to glycosylation. A much broader pattern of expression of the CD44v8/9 domain has been demonstrated in other laboratories using different MAbs directed against epitopes encoded by CD44v8–10 (Terpe *et al.*, 1994; Wielenga *et al.*, 1993), and probably reflects different antibody-binding affinities.

The wide variety of CD44 variant mRNA transcripts detected by RT–PCR in colorectal tumours in this study is striking. Since no pattern of exon usage particularly distinguished tumours by Dukes' stage, a histopathological indicator of colonic tumour progression, the proposal that tumour metastasis can be predicted by RT–PCR of CD44 variants is not tenable. Indeed, some metastatic tumours exhibited no CD44v6 or v7 expression, while some polyps (non-malignant by definition) showed a diverse array of CD44 variant mRNA transcripts containing these exons.

RT–PCR analyses demonstrate the presence of a heterogeneous group of transcripts containing different combinations of exons in the same tumours. These results indicate that a complex array of CD44 variant molecules may exist on the cell surface of many colonic tumours, and this apparent complexity is not readily demonstrable by immunohistochemistry alone. The data shows a tendency for larger transcripts containing additional exons in the more advanced tumours, but these large transcripts may also be found in early cancers and even in some normal colonic mucosal cells. At least eight different transcripts in colorectal tumours were defined by RT–PCR, and there are likely to be additional transcripts not detected by this study. Two transcripts were identified containing the v2 exon which has not yet been previously reported in solid tumours.

Immunohistochemical analyses of colorectal tissue specimens demonstrates that CD44 variant domains encoded by CD44v3, v4/5 and v6 are strongly expressed by at least some tumours from each stage of tumour progression. Moreover, the distribution of CD44 variant positive cells within tumours is heterogeneous, with extent varying from 10 to >90% of malignant cells carrying these epitopes. These results are at odds with a recent report (Mulder *et al.*, 1995) in which a relationship was found between CD44v6 expression and colorectal tumour progression. While the proportion of tumours positive for CD44v6 among high-grade cancers (Dukes' C and D) is similar in both the present study and that of Mulder *et al.* (1995) (81% *vs* 82%), a lower rate of colonic tumour positivity for CD44v6 among low-grade tumours (Dukes' A and B) was found in the latter study (Mulder *et al.*, 1995) (92% *vs* 67%). The reason for this difference is unclear, although the detection of CD44v6-containing mRNA transcripts by RT–PCR in 85% of low-grade colorectal tumours in the present study supports the higher positivity rate for this epitope.

The broad extent of CD44 variant expression found in normal colonic mucosa described in this study has not been previously recognised. While CD44v5, v6 and v9 containing transcripts have been detected previously in normal colonic mucosa by RT–PCR (Wielenga *et al.*, 1993), the respective protein domains have not been demonstrated previously by immunohistochemistry. In the present study, the CD44v6 epitope was detected in as many samples of normal crypt base epithelia as in tumour specimens. CD44 epitopes v3 and v4/5, although more restricted, were expressed in normal colonic mucosa. Since these epitopes were largely confined to the crypt base which is the zone of most rapid cell proliferation, they may play a role in colonic cell proliferation and/or differentiation. Generally, the pattern of CD44 variant exon usage detected by exon-specific RT–PCR in colonic tumours was similar to that seen in the corresponding normal colonic mucosa. This is compatible with the notion that colonic tumour cells may maintain a similar CD44 alternative splicing pattern to the normal mucosa from which they arose, but simply express these epitopes in an overabundance. However, there appear to be exceptions to this rule. In some cases, an entirely different CD44 variant expression pattern or additional exons were detected in the colonic tumour cells compared with the corresponding normal mucosa. In some specimens derived from multiple tumours from the same patients, in which histological differentiation was an obvious variable, different patterns of CD44 variant expression could be detected. This situation is further complicated by the finding that in some specimens CD44 variant expression may be observed on normal colonocytes which are adjacent to the growing tumour margin where, in some cases, the same CD44 variants are not detected in the tumour itself. This curious phenomenon is not explained by current theories of induction of CD44 variant expression in tumours (Hoffman *et al.*, 1993; Jamal *et al.*, 1994), and implies the involvement of as yet unknown trans-acting mechanisms.

This study has not provided evidence for a simple cause-and-effect role for CD44 variant expression and tumour progression. Conversely, the results suggest a complex role for CD44 in colorectal malignancy. The two consistent phenomena demonstrated are a complex array of CD44 splice variants in normal as well as malignant colonic mucosal epithelium, and expression of variable alternatively spliced variant CD44 transcripts in tumours, although particular CD44 variants differ from individual to individual. These findings do not appear to provide support for CD44 variants as a consistent prognostic indicator for colonic tumour progression. There is a need to determine whether CD44 variant expression has a biological role in human epithelial cancers or is simply an epiphenomenon. Studies of the effects of CD44 variant expression on tumour cell invasion and metastasis in model systems will be important for a better understanding of the role of CD44 in tumour progression.

**Acknowledgements**

This work was supported in part by the University of Queensland Special Projects Grant Scheme, the Research and Development Foundation of the Princess Alexandra Hospital, the Research Foundation of the Royal Australasian College of Surgeons, and the Queensland Cancer Fund, Brisbane, Australia.We thank Betty Menzies and the Medical Illustration Department of the Princess Alexandra Hospital for assistance in compilation of the figures and tables. We also thank the surgeons at the Princess Alexandra Hospital for providing the human tissue specimens.

### References

ANTALIS TM AND DICKINSON JL. (1992). Control of plasminogen-activator inhibitor type 2 gene expression in the differentiation of monocytic cells. *Eur. J. Biochem.*, **205**, 203–209.

ARUFFO A, STAMENKOVIC I, MELNICK M, UNDERHILL CB AND SEED B. (1989). CD44 is the principal cell surface receptor for hyaluronate. *Cell*, **61**, 1303–1313.

BROWN TA, BOUCHARD T, ST JOHN T, WAYNER E AND CARTER WG. (1991). Human keratinocytes express a new CD44 core protein (CD44E) as a heparin-sulfate intrinsic membrane proteoglycan with additional exons. *J. Cell. Biol.*, **113**, 207–221.

CHOMCZYNSKI P AND SACCHI N. (1987). Single-step method of RNA isolation by acid guanidinium thiocyanate-phenol-chloroform extraction. *Anal. Biochem.*, **162**, 156–159.

DALL, P, HEIDER K-H, SINN H-P, SKROCH-ANGEL P, ADOLF G, KAUFMANN M, HERRLICH P AND PONTA H. (1995). Comparison of immunohistochemistry and RT–PCR for detection of CD44 variant-expression, a new prognostic factor in human breast cancer. *Int. J. Cancer*, **60**, 471–477.

FEINBERG AP AND VOGELSTEIN B. (1983). A technique for radiolabelling DNA restriction endonuclease fragments to high specific activity. *Anal. Biochem.*, **132**, 6–13.

FINKE LH, TERPE H-J, ZÖRB C, HAENSCH W AND SCHLAG PM. (1995). Colorectal cancer prognosis and expression of exon-v6-containing CD44 proteins (letter). *Lancet*, **345**, 583.

FOX SB, FAWCETT J, JACKSON DG, COLLINS I, GATTER KC, HARRIS AL, GEARING A AND SIMMONS DL. (1994). Normal human tissues, in addition to some tumours, express multiple different CD44 isoforms. *Cancer Res.*, **54**, 4539–4546.

GÜNTHERT U, HOFMANN M, RUDY W, REBER S, ZÖLLER M, HAUBMANN I, MATZKU S, WENZEL A, PONTA H AND HERRLICH P. (1991). A new variant of glycoprotein CD44 confers metastatic potential to rat carcinoma cells. *Cell*, **65**, 13–24.

GUO Y, MA J, WANG J, CHE X, NARULA J, BIGBY M, WU M AND SY M-S. (1994). Inhibition of human melanoma growth and metastasis *in vivo* by anti-CD44 monoclonal antibody. *Cancer Res.*, **54**, 1561–1565.

HART IR, BIRCH M AND MARSHALL JF. (1991). Cell adhesion receptor expression during melanoma progression and metastasis. *Cancer Metast. Rev.*, **10**, 115–121.

HEIDER K-H, HOFMANN M, HORS E, VAN DEN BERG F, PONTA H, HERRLICH P AND PALS S. (1993). A human homologue of the rat metastasis-associated variant of CD44 is expressed in colorectal carcinomas and adenomatous polyps. *J. Cell Biol.*, **120**, 227–233.

HEIDER K-H, DÄMMRICH J, SKROCH-ANGEL P, MÜLLER-HERMELINK H-K, VOLLMERS HP, HERRLICH P AND PONTA H. (1993). Differential expression of CD44 splice variants in intestinal and diffuse-type human gastric carcinomas and normal gastric mucosa. *Cancer Res.*, **53**, 4197–4203.

HOFMANN M, RUDY W, ZÖLLER M, TÖLG C, PONTA H, HERRLICH P AND GÜNTHERT U. (1991). CD44 splice variants confer metastatic behaviour in rats: homologous sequences are expressed in human tumour cell lines. *Cancer Res.*, **51**, 5292–5297.

HOFMANN M, RUDY W, GÜNTHERT U, ZIMMER SG, ZAWADZKI V, ZÖLLER M, LITCHNER RB, HERRLICH P AND PONTA H. (1993). A link between *ras* and metastatic behaviour of tumours cells: *ras* induces CD44 promoter activity and leads to low-level expression of metastastis-specific variants of CD44 in CREF cells. *Cancer Res.*, **53**, 1516–1521.

JACKSON DG, BUCKLEY J AND BELL JI. (1992). Multiple variants of the human lymphocyte homing receptor CD44 generated by insertions at a single site in the extracellular domain. *J. Biol. Chem.*, **267**, 4732–4739.

JALKANEN S, JALKANEN M, BARGATZE R, TAMMI M AND BUTCHER EC. (1988). Biochemical properties of glycoproteins involved in lymphocyte recognition of high endothelial venules in man. *J. Immunol.*, **141**, 1615–1623.

JALKANEN S, JOENSUU H, SÖDERSTRÖM KO AND KLEMI P. (1991). Lymphocyte homing and clinical behaviour of non-Hodgkin's lymphoma. *J. Clin. Invest.*, **87**, 1835–1840.

JAMAL HH, CANO-GAUCI DF, BUICK RN AND FILMUS J. (1994). Activated *ras* and *src* induced CD44 overexpression in rat intestinal epithelial cells. *Oncogene*, **9**, 417–423.

KIM H, YANG X-L, ROSADA C, HAMILTON SR AND AUGUST JT. (1994). CD44 expression in colorectal adenomas is an early event occurring prior to K-ras and p53 gene mutation. *Arch. Biochem. Biophys.*, **310**, 504–507.

KOOPMAN G, HEIDER K-H, HORST E, ADOLF GR, VAN DEN BERG F, PONTA H, HERRLICH P AND PALS ST. (1993). Activated human lymphocytes and aggressive non-Hodgkins lymphomas express a homologue of the rat metastasis-associated variant CD44. *J. Exp. Med.*, **177**, 897–904.

MATSUMURA Y AND TARIN D. (1992). Significance of CD44 gene products for cancer diagnosis and disease evaluation. *Lancet*, **340**, 1053–1058.

MULDER JWR, WIELENGA VJM, POLAK MM, VAN DEN BERG FM, ADOLF GR, HERRLICH P, PALS ST AND OFFERHAUS GJA. (1995). Expression of mutant p53 protein and CD44 variant proteins in colorectal tumorigenesis. *Gut*, **36**, 76–80.

RUDY W, HOFMANN M, SCHWARTZ-ALBIEZ R, ZÖLLER M, HEIDER K-H, PONTA H AND HERRLICH P. (1993). The two-major CD44 proteins expressed on a metastatic rat tumour cell line are derived from different splice variants: each one individually suffices to confer metastatic behaviour. *Cancer Res.*, **53**, 1262–1268.

SCREATON GR, BELL MV, JACKSON DG, CORNELIS FB, GERTH U AND BELL JI. (1992). Genomic structure of DNA encoding the lymphocyte homing receptor CD44 reveals at least 12 alternatively spliced exons. *Proc. Natl Acad. Sci. USA*, **89**, 12160–12164.

SCREATON GR, BELL MV, BELL JI AND JACKSON DG. (1993). The identification of a new alternative exon with highly restricted tissue expression in transcripts encoding the mouse Pgp-1 (CD44) homing receptor. *J. Biol. Chem.*, **268**, 12235–12238.

SEITER S, ARCH R, REBER S, KOMITOWSKI D, HOFMANN M, PONTA H, HERRLICH P, MATZKU S AND ZÖLLER M. (1993). Prevention of tumor metastasis formation by anti-variant CD44. *J. Exp. Med.*, **177**, 443–455.

SOUTHGATE J, TREJDOSIEWICZ LK, SMITH B AND SELBY PS. (1995). Patterns of splice variant CD44 expression by normal human urothelium *in situ* and *in vitro* and by bladder carcinoma cell lines. *Int. J. Cancer*, **62**, 449–456.

STAMENKOVIC I, AMIOT M, PESANDO JM AND SEED B. (1989). A lymphocyte molecule implicated in lymph node homing is a member of the cartilage link protein family. *Cell*, **56**, 1057–1062.

STAMENKOVIC I, ARUFFO A, AMIOT M AND SEED B. (1991). Human keratinocyptes express a new CD44 core protein (CD44E) as a heparin-sulfate intrinsic membrane proteoglycan with additional exons. *EMBO J.*, **10**, 343–348.

SY MS AND GUO YJ. (1991). Distinct effects of two CD44 isoforms on tumour growth *in vivo*. *J. Med. Exp.*, **174**, 859–866.

TAKADA M, YAMAMOTO M AND SAITOH Y. (1994). The significance of CD44 in human pancreatic cancer: I. High expression of CD44 in human pancreatic adenocarcinoma. *Pancreas*, **9**, 748–752.

TERPE H-J, STARK H, PREHM P AND GÜNTHERT U. (1994). CD44 variant isoforms are preferentially expressed in basal epithelia of non-malignant human fetal and adult tissues. *Histochemistry*, **101**, 79–89.

WIELENGA VJM, HEIDER K-H, OFFERHAUS GJA, ADOLF GR, VAN DEN BERG FM, PONTA H, HERRLICH P AND PALS ST. (1993). Expression of CD44 variant proteins in human colorectal cancer is related to tumor progression. *Cancer Res.*, **53**, 4754–4756.